# Identifying Risk Factors for Complicated Post-operative Course in Tetralogy of Fallot Using a Machine Learning Approach

**DOI:** 10.3389/fcvm.2021.685855

**Published:** 2021-07-22

**Authors:** Jennifer A. Faerber, Jing Huang, Xuemei Zhang, Lihai Song, Grace DeCost, Christopher E. Mascio, Chitra Ravishankar, Michael L. O'Byrne, Maryam Y. Naim, Steven M. Kawut, Elizabeth Goldmuntz, Laura Mercer-Rosa

**Affiliations:** ^1^Data Science and Biostatistics Unit, Department of Biomedical and Health Informatics, Children's Hospital of Philadelphia, Philadelphia, PA, United States; ^2^Department of Biostatistics, Epidemiology & Informatics, University of Pennsylvania, Philadelphia, PA, United States; ^3^Department of Pediatrics, Children's Hospital of Philadelphia, Philadelphia, PA, United States; ^4^School of Public Health, Brown University, Providence, RI, United States; ^5^Division of Cardiothoracic Surgery, Department of Surgery, Children's Hospital of Philadelphia, University of Pennsylvania Perelman School of Medicine, Philadelphia, PA, United States; ^6^Division of Cardiology, Department of Pediatrics, Children's Hospital of Philadelphia, University of Pennsylvania Perelman School of Medicine, Philadelphia, PA, United States; ^7^Leonard Davis Institute and Center for Cardiovascular Outcomes, Quality, and Evaluative Research, University of Pennsylvania, Philadelphia, PA, United States; ^8^Departments of Anesthesiology, Critical Care Medicine and Pediatrics, Children's Hospital of Philadelphia, University of Pennsylvania Perelman School of Medicine, Philadelphia, PA, United States; ^9^Departments of Medicine and Epidemiology, University of Pennsylvania Perelman School of Medicine, Philadelphia, PA, United States

**Keywords:** tetralogy of Fallot, cardiopulmonary bypass, global longitudinal strain imaging, outcome, congenital heart defect

## Abstract

**Introduction:** Tetralogy of Fallot (TOF) repair is associated with excellent operative survival. However, a subset of patients experiences post-operative complications, which can significantly alter the early and late post-operative course. We utilized a machine learning approach to identify risk factors for post-operative complications after TOF repair.

**Methods:** We conducted a single-center prospective cohort study of children <2 years of age with TOF undergoing surgical repair. The outcome was occurrence of post-operative cardiac complications, measured between TOF repair and hospital discharge or death. Predictors included patient, operative, and echocardiographic variables, including pre-operative right ventricular strain and fractional area change as measures of right ventricular function. Gradient-boosted quantile regression models (GBM) determined predictors of post-operative complications. Cross-validated GBMs were implemented with and without a filtering stage non-parametric regression model to select a subset of clinically meaningful predictors. Sensitivity analysis with gradient-boosted Poisson regression models was used to examine if the same predictors were identified in the subset of patients with at least one complication.

**Results:** Of the 162 subjects enrolled between March 2012 and May 2018, 43 (26.5%) had at least one post-operative cardiac complication. The most frequent complications were arrhythmia requiring treatment (*N* = 22, 13.6%), cardiac catheterization (*N* = 17, 10.5%), and extracorporeal membrane oxygenation (ECMO) (*N* = 11, 6.8%). Fifty-six variables were used in the machine learning analysis, of which there were 21 predictors that were already identified from the first-stage regression. Duration of cardiopulmonary bypass (CPB) was the highest ranked predictor in all models. Other predictors included gestational age, pre-operative right ventricular (RV) global longitudinal strain, pulmonary valve Z-score, and immediate post-operative arterial oxygen level. Sensitivity analysis identified similar predictors, confirming the robustness of these findings across models.

**Conclusions:** Cardiac complications after TOF repair are prevalent in a quarter of patients. A prolonged surgery remains an important predictor of post-operative complications; however, other perioperative factors are likewise important, including pre-operative right ventricular remodeling. This study identifies potential opportunities to optimize the surgical repair for TOF to diminish post-operative complications and secure improved clinical outcomes. Efforts toward optimizing pre-operative ventricular remodeling might mitigate post-operative complications and help reduce future morbidity.

## Introduction

Surgical reconstruction for Tetralogy of Fallot (TOF) is associated with low mortality but with significant long-term morbidity resulting from residual lesions and need for reinterventions (1–14). There is also evidence that post-operative complications resulting in a prolonged hospitalization can result in ongoing morbidity (3, 15, 16). Therefore, reducing perioperative risk and complications might be beneficial for patients and their families. Although traditional risk factors have been reported, including history of premature birth, neonatal surgical repair, and a less-favorable TOF anatomy (pulmonary atresia with multiple aortopulmonary collateral vessels, for example), the breadth of risk factors has not been fully explored with traditional methods. Studying risk factors for post-operative complications using a machine learning approach might unveil risk factors not previously considered, or combinations of factors without the limitation of collinearity in classic multivariable regression models. Identifying patients at high risk for complications will allow for strategies that might improve outcomes. We hypothesized that post-operative complications are not infrequent after TOF surgery and sought to investigate risk factors for such complications using a machine learning method. Prediction of post-operative cardiac complications is important, as mitigating complications could improve overall outcomes.

## Methods

### Study Design and Participants

This study utilized data from a single-center prospective cohort study of 162 TOF patients recruited at the Children's Hospital of Philadelphia from March 2012 to May 2018 described previously (17). The study was approved by the Institutional Review Board. Inclusion criteria included TOF repair performed in a single stage (including relief of right ventricular outflow tract obstruction if necessary, and closure of the ventricular septal defect) or preceded by a palliative procedure (for example, aortopulmonary shunt, ductal stent). Those who underwent a palliative procedure alone, and/or unifocalization of the pulmonary arteries and right ventricle to pulmonary artery conduit placement followed by later closure of the ventricular septal defect, and TOF completion after 2 years of age were excluded. Aortopulmonary collaterals were diagnosed by cardiac catheterization and/or cardiac magnetic resonance imaging.

Data collection included clinically indicated pre-operative echocardiograms closest to the date of surgery and daily review of medical records for patient and perioperative characteristics. Measures of right ventricular (RV) function included tissue Doppler (peak tricuspid E, A, and e' velocities, and the E/e' ratio, all of which are indicative of RV diastolic function), and measures of RV systolic function, including the tissue Doppler peak tricuspid S' velocity, RV fractional area change, RV global longitudinal strain, and RV strain rate (18). RV fractional area change was derived by tracing the endocardial border of the RV at end-diastolic and end-systole on apical four chamber views. The area change was calculated by subtracting the end-systolic area from the end-diastolic area, over the end-diastolic area. The outcome was post-operative cardiac complications. We identified the number of post-operative cardiac complications that occurred between TOF repair and hospital discharge or death (whichever came first), including the following: mediastinal exploration, delayed sternal closure, pleural effusion requiring chest tube, pericardial effusion requiring drainage, arrhythmia requiring treatment, cardiac catheterization, cardiac reoperation, cardiac arrest, and cardiopulmonary resuscitation, need for ECMO, unanticipated cardiac procedures, and pacemaker placement. A complete list of cardiac complications is detailed in Supplementary Table 1. Cardiac catheterizations were included as complications because this procedure is not anticipated after the uncomplicated TOF repair. Indications for cardiac catheterization were identified and included persistent/significant hypoxemia, low cardiac output/persistent ventricular dysfunction, and evidence of significant residual lesions on echocardiogram (hemodynamically significant ventricular septal defects, branch pulmonary artery stenosis). Indications for chest drainage included greater than moderate pleural effusion with failure of medical management. All post-operative echocardiograms were performed as part of the study protocol between post-operative days 2 and 5.

Patients were included in the study when TOF surgery was scheduled; thus, if patients died prior to completion of surgical repair or died after a palliation and did not reach completion of repair, they were not included in the study.

### Analysis

A comprehensive list of variables was generated including patient, operative and post-operative factors. Patient factors included demographics (race, ethnicity, maternal education, and age at repair), birth history (birth weight and gestational age), pre-operative oxygen saturation, use of prostaglandin, and number of hospital admissions. Cardiac diagnosis was confirmed with offline review of echocardiograms, and offline measurements were made of the pulmonary valve, pulmonary arteries, and measures of right ventricular function. We confirmed a genetic diagnosis including knowledge of 22q11.2 deletion status for all patients and collected operative characteristics (duration of cardiopulmonary bypass (CPB) and aortic cross clamp, number of CPB runs, use of deep hypothermic circulatory arrest, lowest pH, temperature and hematocrit during bypass, and type of surgical repair) and details of the surgical repair.

#### Missing Data

We included variables that occurred before or around the time of surgical repair in our analytic models. Immediate post-operative oxygen saturation upon arrival to the intensive care unit was included as a predictor. All variables included in the analysis had <10% missing data. Missing data were imputed using a fully conditional specification method with 10 imputations for continuous and categorical covariates using the SAS Procedure Proc MI (19). All analytic [regression-based or gradient-boosted models (GBM)] models were run individually on each imputed dataset.

Pre-operative echocardiograms were clinically obtained, as they occurred prior to patient enrollment.

#### Main Analytic Models: Gradient-Boosted Models

Gradient-boosted quantile regression models were used to identify predictors of number of post-operative cardiac complications at the 50th percentile (20) (Supplementary Material). For each imputed dataset, 80% of the data were used as training data for model development and 20% as the holdout testing set for model evaluation. On each imputed training dataset, a 10-fold cross-validated (CV) GBM was performed using 5,000 gradient-boosted trees and determined the optimal number of trees which minimized the cross validated root mean square error (RMSE). Each GBM provided a relative importance score for all of the variables included in the model and the relative importance scores across 10 multiply-imputed datasets were averaged. Model fit in the testing data was assessed by fitting the model from the training dataset to predict values in the testing data. Average RMSE were calculated across the 10 imputed datasets for the training data and testing data. Lower RMSE values indicated better model fit. GBMs were run using the GBM package in R 3.4.3 (R Development Core Team, Vienna, Austria) (21).

We compared both the average RMSE for the training data and average RMSE for the test data from the boosted quantile regression to corresponding average RMSE for training and test data obtained from stepwise linear regression, a commonly used approach for prediction models with a continuous outcome.

To test the importance of a multistage modeling approach to obtain the final set of predictors, we used a two-stage approach running the GBM with and without a multivariate adaptive regression spline model (MARS) to select a subset of clinically meaningful predictors. A negative binomial distribution was assumed for the outcome (Supplementary Material). MARS provided the relative importance of each predictor identified in the final model (22). Of the 56 predictors included in the MARS model, we chose the top 50% predictors (*N* = 21) across the 10 imputed datasets as most important and ran the second stage GBMs using these predictors. We averaged the relative importance of the variables in the 10 imputed datasets to obtain an overall relative importance for each predictor.

#### ROC Curve Analysis of Cardiopulmonary Bypass Time

Because CPB time emerged as the single strongest predictor of post-operative complications, we conducted receiver-operating characteristic (ROC) curve analysis of the number of post-operative complications (categorical outcome with levels 0 complication, one to two complications, and three or more complications) to test how well CPB duration predicts this outcome. We split the data 1:1 into test and training datasets and performed univariable multinomial logistic regression to quantify the predictive ability of total CPB time. In the training data, we used the Youden Index to determine the optimal cut-off value for total CPB Time since this index maximized sensitivity and specificity, and then scored the data in the test dataset and obtained the area under the curve (AUC) values using the SAS Macro MultAUC (available at http://support.sas.com/kb/64/addl/fusion_64029_12_multauc.sas.txt). We provide an overall AUC across the three levels of the outcomes as well as AUC values comparing pairs of outcomes. Bootstrapped 95% confidence intervals for the AUC values were the 2.5th and 97.5th percentiles obtained from 1,000 bootstrapped samples. Since there were no missing data for either the predictor or outcome, we ran the analysis using the full sample of 162 patients.

### Sensitivity Analyses

#### Gradient-Boosted Poisson Models

Sensitivity analysis using gradient-boosted Poisson regression models was performed on the subset of 43 patients with at least one complication to examine how the occurrence of no post-operative complication (zero outcome) would affect results. The average relative importance of predictors run using all 56 predictors and using the 21 predictors obtained from using a first-stage regression from the main analysis are presented.

#### Gradient-Boosted Quantile Regression Models Without Total CPB Time

In order to examine if a model without the most important predictor would significantly alter the results, we ran gradient boosted quantile regression models using all predictors except the most important predictor, total CPB time, and averaged the variable importance scores across the 10 imputed datasets.

## Results

### Characteristics of the Patient Population

The families of 188 children with TOF were approached and 162 (86%) consented. Demographic, pre-operative, operative, and post-operative descriptive data for the 162 subjects is found in Table 1. In short, more patients were male (62.7%) and white (64.2%). There were 28 patients with a prior palliative procedure, including: BT shunt (*n* = 17), pulmonary valve balloon dilation (*n* = 3), RVOT stent (*n* = 3), PDA stent (*n* = 3), and central shunt (*n* = 2). Mean age of TOF repair was 3.9 months (standard deviation (SD) 2.9 months. Ninety-five patients (58.6%) received a transannular patch as part of the surgical repair. Deep hypothermic circulatory arrest (DHCA) was used in 12 patients (7.40%). There were nine patients (5.56%) with aortopulmonary collaterals, six of whom underwent unifocalization. Neonatal (≤30 days of age) complete TOF repair occurred in 24 patients (14.8%). At least one post-operative cardiac complication occurred in 43 patients (26.5%). Post-operative complications included arrhythmia requiring treatment (*N* = 22; 13.6%), cardiac catheterization (*N* = 17, 10.5%), ECMO (*N* = 11, 6.8%), mediastinal exploration (*N* = 9, 5.6%), and pleural effusion requiring a chest tube (*N* = 8, 4.9%) (Table 2). Seven patients underwent reoperation, or experienced cardiac arrest/cardiopulmonary resuscitation, delayed sternal closure, any other cardiac procedure, pericardiocentesis, and pacemaker placement. The earliest complication occurred on the day of surgery (post-operative day 0), the latest occurred on post-operative day 21. An arrhythmia requiring treatment occurred on post-operative day 0 or 1 for most patients (19 out of 22), whereas a cardiac catheterization occurred anywhere from post-operative day 0 to post-operative day 21. Non-cardiac events occurred in 13 patients (8.0%) (Table 2). On post-operative echocardiograms, most patients (84%) had a patent foramen ovale, of which 44% had right to left shunting and 46% had bidirectional shunting.

**Table 1:** Patient and operative and post-operative characteristics.

**Patient and pre-operative characteristics**	
Age at repair (months), mean (SD)	3.9 (2.90)
Age at repair for those with no prior palliation, mean (SD)	3.5 (2.88)
Age at repair for those with prior palliation, mean (SD)	5.9 (2.03)
Male, *n* (%)	101 (62.73%)
Race/ethnicity non-hispanic white, *n* (%)	104 (64.20%)
Birth weight (kg), mean (SD)	2.91 (0.78)
Gestational age (weeks), mean (SD)	37.50 (2.83)
Prenatal diagnosis, *n* (%)	117 (72.22%)
**Pulmonary valve anatomy**, ***n*** **(%)**	
Stenosis	133 (82.10%)
Atresia	25 (15.43%)
Absent	4 (2.47%)
Aortopulmonary collaterals, *n* (%)	9 (5.56%)
Genetic diagnosis, *n* (%)	41 (25.31%)
Extracardiac malformation, *n* (%)	74 (45.68%)
Pre-operative interventions, *n* (%)	28 (17.28)
Blalock-Taussig shunt	17 (10.49)
Pulmonary valve balloon dilation	3 (1.85)
Right ventricular outflow tract stent	3 (1.85)
PDA stent	3 (1.85)
Central aortopulmonary artery shunt	2 (1.24)
**Operative characteristics, mean (SD)**	
**Access for intracardiac repair of TOF, ***n*** (%)**	
Transatrial	56 (34.57)
Transventricular	27 (16.67)
Transatrial and transventricular	79 (48.77)
**Foramen ovale, ***n*** (%)**	
Closed	40 (24.69)
Left open	108 (66.67)
Created	14 (8.64)
**Outflow tract repair**, ***n*** **(%)**	
Transannular patch	95 (58.64)
Non-transannular patch	10 (6.17)
Right ventricle to pulmonary artery conduit	18 (11.11)
Pulmonary valvotomy	16 (9.88)
VSD closure only (no outflow tract augmentation)	23 (14.2)
Number of CPB runs, mean (SD)	1.15 (0.52)
Patients with repeated CPB runs >1, *n* (%)	17 (10.49%)
Total CPB time (min), mean (SD)	66.35 (38.76)
Total aortic cross-clamp time (min), mean (SD)	46.26 (24.70)
DHCA use, *n* (%)	12 (7.40)
DHCA time (minutes), mean (SD)	25.67 (12.91)
Lowest PH on CPB, mean (SD)	7.31 (0.074)
Lowest esophageal temperature (centigrade), mean (SD)	32.89 (5.63)
Lowest hematocrit on CPB, mean (SD)	34.25 (4.67)
**Post-operative characteristics, median (25th and 75th percentiles)**	
Length of hospital stay, days	7 (5, 12)
Number of days on oxygen	3 (2, 6)
Days of chest tube	1 (1, 3)
Days of central line	2 (0, 5)
Number of inotropes	2 (1, 7)
Number of cardiac medications	1 (1, 2)
Number of non-cardiac medications	0.5 (0, 1)
Number of all medications	2 (1.5, 3)
Days on inotropes	2 (1, 5)
Prolonged hospital stay >30 days, *n* (%)	20 (12.3)

*CPB, cardiopulmonary bypass; DHCA, deep hypothermic circulatory arrest; ECMO, extracorporeal membrane oxygenation*.

**Table 2:** Post-operative complications.

**At least 1 post-operative cardiac complication, ***N*** (%)**	43 (26.54%)
**Type of cardiac complication**	
Arrhythmia requiring treatment	22 (13.58%)
Cardiac catheterization	17 (10.49%)
ECMO	11 (6.79%)
Mediastinal exploration	9 (5.56%)
Pleural effusion requiring chest tube	8 (4.94%)
Chest left open (delayed sternal closure)	7 (4.32%)
Reoperation	7 (4.32%)
Cardiac arrest and CPR	6 (3.70%)
Other cardiac procedure	5 (3.09%)
Pericardial effusion with pericardiocentesis	1 (0.62%)
Pacemaker placement	1 (0.62%)
**Post-operative day of arrhythmia requiring treatment**	
0	15 (68.18%)
1	4 (18.18%)
2–5	3 (13.61%)
**Post-operative day of catheterization (*N* = 17)**	
0	3 (17.65%)
3	5 (29.41%)
6–21	9 (52.90%)
**Post-operative day of any cardiac complication (*N* = 43)**	
0	28 (65.12%)
1	6 (13.95%)
2	1 (2.33%)
3	2 (4.65%)
4	2 (4.65%)
>7	4 (9.31%)
**Post-operative non-cardiac events, *N* (%)**	13 (8.00%)
G-Tube placement	3 (1.85%)
Pneumothorax requiring treatment	3 (1.85%)
Seizure requiring treatment	2 (1.23%)
Superficial wound infection	2 (1.23%)
Urinary tract infection	2 (1.23%)

*ECMO, extracorporeal membrane oxygenation; CPR, cardiopulmonary resuscitation. ECMO, bronchoscopy, airway malacia, necrotizing enterocolitis, sepsis, and vocal cord paralysis occurred in one patient each*.

### Missing Data

Missing data was identified in pre-operative echocardiographic variables that are used to assess RV systolic function, including RV longitudinal strain and fractional area change in 9.9% of patients. All other predictors had <9% missing data.

### Gradient-Boosted Quantile Regression Models

All predictors are presented from highest to lowest in terms of average relative importance (Table 3). All models identified total CPB time as the highest ranked predictor, followed by pre-operative RV global longitudinal strain, immediate post-operative arterial oxygen level, aortic cross-clamp time, pulmonary valve Z-score, gestational age, lowest esophageal/nasal temperature during TOF repair, left pulmonary artery Z-score, right pulmonary artery Z-score, lowest hematocrit on CPB, use of oxygen at the time of TOF repair, race, pre-operative RV fractional area change, prenatal diagnosis of TOF, and pre-operative oxygen saturation. GBM results run after first-stage regressions are presented in Table 4 (21 predictors). These results reinforce the same findings identified using all 56 predictors.

**Table 3:** GBM including all variables (no first stage regression; *N* = 56 variables).

**Predictor (ranked in descending strength)**	**Average importance**
Total CPB time	54.71
Global longitudinal strain–RV % (pre-operative)	7.50
Partial pressure of O_2_ (paO_2_) immediately after TOF repair	4.06
Total aortic cross-clamp time (min)	3.86
Pulmonary valve annulus Z-score	3.71
Gestational age (weeks)	2.46
Lowest esophageal/nasal temperature during CPB	2.20
Left pulmonary artery Z-score	2.05
Right pulmonary artery Z-score	1.72
Lowest hematocrit during CPB	1.71
Room air upon TOF repair	1.63
Race/ethnicity	1.63
RV factional area change (pre-operative)	1.62
Prenatal diagnosis of TOF	1.27
Oxygen saturation (pre-operative)	1.27
Ventriculotomy used for TOF repair	1.03
Lowest PH on CPB	0.94
Birth weight, kg	0.90
Age at time of surgery (months)	0.84
Maternal education = 13–15 years	0.68
Aortopulmonary collaterals	0.65
Any extracardiac malformation	0.50
Endocardial cushion defect	0.38
Surgeon #2 (3 different surgeons)	0.36
Pulmonary valve stenosis (vs. atresia, or absent)	0.32
Foramen ovale close during TOF repair	0.21
Right aortic arch	0.18
Transatrial assess for TOF repair	0.17
Genetic diagnosis	0.17
BT shunt prior to TOF repair (pre-operative intervention)	0.16
Maternal education = 16 or more years	0.14
Any extracardiac anomaly	0.13
No pre-operative interventions	0.10
Admitted from outside hospital (vs. home)	0.10
Number of CPB runs	0.10
Surgeon #3 (3 different surgeons)	0.08
Race/ethnicity = non-hispanic black	0.08
Transannular patch used for TOF repair	0.07
Feeding status at admission = NPO	0.05
Deep hypothermic circulatory arrest	0.05
Created foramen ovale during TOF repair	0.04
Admitted from another unit (vs. home)	0.03
Plasty of pulmonary arteries during TOF repair	0.03
Continuous pulmonary arteries	0.02
Genetics consultation obtained	0.02
Coronary = single coronary/LAD from RCA/dual LAD	0.02
Pulmonary valvotomy (for outflow tract repair)	0.02
RV-PA conduit (for outflow tract repair)	0.02
Non-transannular patch (for outflow tract repair)	0.01
Prostaglandin use at admission	0.01
Natural conception (vs. assisted reproductive methods)	0.01
Race/ethnicity = hispanic	0.01
Sex	0.00
Neonatal repair (vs. repair at a later time)	0.00
Patch extended to LPA during TOF repair	0.00
Patient was readmitted prior to this admission	0.00

*BT, Blalock Taussig; LAD, left anterior descending; RCA, right coronary artery; RV-PA, right ventricle to pulmonary artery; LPA, left pulmonary artery*.

**Table 4:** GBM run after first-stage regressions (*N* = 21 variables).

**Predictor (ranked in descending strength)**	**Average importance**
Total CPB time	55.23
Pulmonary valve annulus Z-score	7.29
Global strain–RV % (pre-operative)	6.60
Partial pressure of O_2_ immediately after TOF repair	4.37
Total aortic cross-clamp time (min)	4.19
Gestational age (weeks)	3.19
Left pulmonary artery Z-score	3.02
Right pulmonary artery Z-score	2.17
Lowest esophageal/nasal temperature in OR	2.13
RV fractional area change (pre-operative)	1.96
Air type = room air upon admission for TOF repair	1.66
Race/ethnicity = non-hispanic other	1.57
Lowest hematocrit on CPB	1.56
Birth weight, kg	1.11
Lowest PH on CPB	1.04
Age at time of surgery (months)	0.87
Maternal education = 13–15 years	0.69
Endocardial cushion effect	0.55
Genetics consults done	0.48
Number of CPB runs	0.30
Patch extended to LPA during TOF repair	0.01

### Predictive Accuracy

Compared with linear regression (data not shown), the GBMs had, on average, smaller RMSE in both the training and test data, suggesting that the GBMs more accurately predicted post-operative complications. Using the analyses with all 56 predictors, the RMSE for the GBM training and test data was 0.71 and 1.04, respectively, compared with average RMSE values 1.68 (training) and 2.42 (test) from linear regression.

### ROC Curve Analysis

ROC analysis was conducted using the total CPB time as the predictor and a three-level outcome: no complications, one or two complications, and three or greater complications. In the training data, the overall AUC was 0.77, 95% CI (0.64, 0.85) and in the test data, the AUC was 0.71 with 95% CI (0.61, 0.84). The best discriminatory capability of CPB time was the ROC comparing 0 vs. 3 complications for training and test data [AUC: 0.94 (95% CI: 0.77, 1), AUC: 0.88 (95% CI: 0.77, 1), respectively]. Other calculated AUC are detailed in Table 5.

**Table 5:** CPB as a predictor of number of post-operative complications.

	**Overall AUC (95% CI)**	**0 vs. 1 or 2 AUC (95% CI)**	**0 vs. ≥3 AUC (95% CI)**	**1 or 2 vs. ≥3 AUC (95% CI)**
Training data	0.77 (0.64, 0.85)	0.63 (0.46, 0.77)	0.94 (0.77, 1)	0.75 (0.51, 0.98)
Test data	0.71 (0.61, 0.84)	0.56 (0.40, 0.74)	0.88 (0.77, 1)	0.70 (0.5, 0.96)

### Sensitivity Analyses

#### Gradient-Boosted Poisson Models

Sensitivity analysis was conducted with gradient-boosted Poisson models using the subset of the sample that had at least one post-operative cardiac complication (*N* = 43 patients). These models were run using all 56 predictors (no first stage regression) as well as only using the 21 variables deemed important from the first-stage regression in the main analysis.

The gradient boosted-based Poisson models identified total CPB time as the highest ranked predictor followed by age at the time of surgery, and other similarly ranked predictors, including: pre-operative fractional area change, right pulmonary artery Z-score, admission from outside hospital (vs. home), left pulmonary artery Z score, aortic cross-clamp time, and prostaglandin use at the time of TOF repair, confirming the conclusions reached from the gradient boosted quantile regression models (Supplementary Tables 2, 3). The average RMSE for the boosted Poisson models using all 56 predictors and 21 predictors was considerably larger than the average RMSE for test and training data using the gradient boosted quantile models (4.18 and 4.21, respectively).

#### Gradient-Boosted Quantile Regression Models Without Total CPB Time

GBMs run without CBP as a predictor identified five highly ranked predictors of number of post-operative complications, from most to least important: lowest hematocrit on CPB, left pulmonary artery Z-Score, partial pressure of O_2_ (paO_2_) after TOF repair, total aortic cross-clamp time, and presence of aortopulmonary collaterals. The remaining 50 predictors had no importance in any of the models that did not include CPB time.

## Discussion

In this study, we applied a machine learning approach to identify risk factors for post-operative complications after TOF repair. This approach allowed for identification of a broad set of predictors, irrespective of collinearity. Our main findings indicate that post-operative cardiac complications were not infrequent and tended to occur early after TOF repair (on the same day of or on the day after surgery). The highest ranked predictors encompassed both pre- and intraoperative factors. Findings were similar in sensitivity analyses limited to the subset of patients that had at least one post-operative complication.

Post-operative complications were common in our study and are important because they can significantly alter the early and late post-operative course after TOF repair, with prolonged length of hospital stay as well as long-term morbidity and increased risk of death, as shown in other studies (2, 3, 23–25). Similar to our findings, a recent study by van den Bosch et al. reported that post-operative complications occurred in a quarter (25.6 %) of patients undergoing TOF repair with a transannular patch. Additionally, in long-term follow-up to late adolescence, a post-operative complication had twice the hazard of a late event, which included death, pulmonary valve replacement, reoperations, cardiac catheterizations, and hospitalizations, even in the group that underwent elective TOF repair (2).

To our knowledge, machine learning methods have not yet been used to predict post-operative complications in patients with TOF but have been used in studies in other congenital heart defects. In a study of infants with single-ventricle physiology, naïve Bayesian network models were used to predict critical events that occurred 1 to 8 h prior to the complication (26). The objective in our study was to apply machine learning methods to identify a set of predictors of post-operative complications, rather than using conventional approaches like linear regression, which we found yielded less accurate predictions and is unable to provide a ranking of predictors.

The GBM approach with and without a first-stage regression consistently identified total CPB time as the most important predictor. This finding was perhaps not surprising given that a prolonged CPB during surgical repair for TOF likely indicates a technically difficult procedure, complex TOF anatomy, and/ or residual lesions that resulted in >1 CPB run, as shown by others (27). The correlation between CPB duration and post-operative complications is well-known and not exclusive to TOF. In addition, CPB results in a systemic inflammatory response which is associated with major morbidities including renal and neurologic injury, reoperation, cardiac arrest and need for post-operative mechanical circulatory support in as many as 20–30% of cases (28–32).

Our sensitivity analysis without the most important predictor still reinforced the main findings of the importance of total CPB time. Interestingly, when CPB was removed from the GBM, the predictors identified by the model were still related to CPB, such as hematocrit during CPB, LPA size (requiring LPA arterioplasty, possibly resulting in longer CPB), partial pressure of oxygen in arterial blood after TOF repair (possibly representing atrial right to left shunting in a stiff right ventricle after a long CPB run, which was present in most patients in our study), aortic cross-clamp duration (a part of CPB), and presence of aortopulmonary collaterals, which can introduce technical difficulty during surgery and prolong the use of CPB. Furthermore, collateral vessels can result in bleeding (flooding) in the surgical field, requiring blood to be returned to the extracorporeal circuit, as shown by others (33). Therefore, CPB is likely a strong indicator of technical challenges and remains a strong indicator of post-operative complications.

To our knowledge, this is the first time that pre-operative RV function has been linked to post-operative cardiac complications in TOF patients. This adds novelty to our findings and reiterates the concept that machine learning allows for identification of non-traditional factors, or at least factors that have not been considered in standard regression analyses, such as pre-operative RV global longitudinal strain. Other parameters of RV function included tricuspid S' velocity, RV fractional area change, diastolic RV function measured as E/e' ratio, and E and A velocities. Pre-operative RV fractional area change was as a predictor but ranked lower than the pre-operative RV global longitudinal strain. This is likely due to the fact that deformation parameters, such as strain, are more sensitive to detect subclinical changes in function before overt changes can be seen with geometric or volumetric methods, such as fractional area change or ejection fraction, as shown in infants with single right ventricles and adults after heart transplant (34, 35). While qualitative RV systolic function may be seemingly preserved pre-operatively, significant pre-operative RV remodeling with hypertrophy and subclinical dysfunction with altered myocardial deformation may occur and therefore, quantitative pre-operative assessment is important. In a study of post-operative outcomes in TOF, pre-operative factors such as severity of pulmonary stenosis and higher C-reactive protein levels were associated with complicated post-operative course, but pre-operative ventricular functional parameters were not taken into account (27). Post-operative RV diastolic dysfunction has been previously studied, and when present early after TOF, it is associated with longer post-operative hospitalization (36, 37).

Other predictors identified in our analysis include race, maternal education, and other anatomic and intraoperative factors. Anatomic factors, such as the size of the pulmonary valve and the pulmonary arteries, and continuity of the pulmonary arteries are known to represent a more severe TOF phenotype, and therefore the association with post-operative complications is not surprising (3, 38). Intraoperative factors, such as low pH, temperature, and hematocrit during bypass were also predictors of post-operative complications, suggesting that tight control of these parameters during CPB is warranted. Not only are these factors associated with post-operative complications but they have also been shown to correlate to neurodevelopmental outcomes at 1 year of age in patients with TOF and other heart defects (39). Finally, patient factors including birth weight and maternal education, which are not readily modifiable, were associated with post-operative complications. Birth weight could be a surrogate an unrecognized genetic syndrome or of smaller weight at TOF repair, possibly causing a more technically challenging operation. However, low birth weight is associated with long-term neurodevelopmental outcomes irrespectively of the complexity of surgery (40). While to our knowledge the role of maternal education on outcome has not been investigated in TOF, maternal education was a mediator of the relationship between race/ethnicity and increased mortality in infants with other congenital heart defects as shown in a population-based study in 2018 (41). These important factors certainly warrant further study.

In summary, we identified a set of predictors of post-operative complications after surgical repair for TOF. These initial observations should be used in future analyses to derive clinical prediction rules for post-operative complications that can be incorporated into TOF pre-surgical planning. Strategies targeting modifiable factors could include, for example, modifying the age at complete repair by palliating the neonate with a stent in the ductus arteriosus, altering the intraoperative management with optimization of temperature, pH, and hematocrit, and more aggressive use of perioperative drugs to support RV systolic function (24, 42, 43).

Our study has some limitations, as well as some notable strengths. This was a single-center study; therefore, the sample size was limited by the number of TOF repairs performed at our institution. However, although limited to a single referral center, our results may yet be generalizable to similar tertiary centers. Future multicenter studies would ideally integrate hospital-level factors into the analysis. Because only patients that consented to the study are part of this analysis, not all patients operated for TOF in our center were included, possibly resulting in selection bias. However, the consent rate for this study was in excess of 85%. Misclassification bias (for instance, error in the number of post-operative complications) is unlikely due to the prospective nature of the study and careful data collection. A notable strength of the study, prospective data collection resulted in minimal missing data, even though there was missing data from the pre-operative echocardiograms, which were performed for clinical purposes. Although post-operative echocardiograms were obtained as part of the research study, those factors were not included in the study because we could not classify certain variables; for example, if the presence of post-operative right ventricular dysfunction was present, it could have been a predictor of a post-operative complication, or be considered a post-operative complication itself. A future study would ideally also include other predictors such as biomarkers, neighborhood-level characteristics, and clinical measures collected over time. Despite its limitations, this study provides the groundwork for future investigations of post-operative complications in TOF patients.

## Conclusion

Post-operative cardiac complications are prevalent in patients undergoing complete repair for TOF. Duration of CPB remains an important predictor of post-operative complications after TOF repair, perhaps reflecting the combined effects of age at repair and complexity of the presenting anatomy. These results suggest that attention to other perioperative factors and management are likewise important, including attention to pre-operative right ventricular remodeling. Overall, this study identifies potential opportunities to modify surgical strategies for TOF to diminish post-operative complications and secure improved clinical outcomes. Efforts toward optimizing pre-operative ventricular remodeling might mitigate post-operative complications and help reduce future morbidity.

## Data Availability Statement

The raw data supporting the conclusions of this article will be made available by the authors, without undue reservation.

## Ethics Statement

The studies involving human participants were reviewed and approved by Institutional Review Board, Children's Hospital of Philadelphia. Written informed consent to participate in this study was provided by the participants' legal guardian/next of kin.

## Author Contributions

LM-R, EG, and SK contributed to conception and design of the study. GD, LM-R, and JF organized the database. JF, JH, XZ, and LS performed together with JF the statistical analysis. LM-R, CM, CR, MO'B, MN, SK, and EG were involved in the implementation of the study and interpretation of data. LM-R was responsible for the supervision of the study. JF and LM-R wrote the first draft of the manuscript. All authors reviewed and edited all sections of the manuscript and read and approved the submitted manuscript version.

## Conflict of Interest

The authors declare that the research was conducted in the absence of any commercial or financial relationships that could be construed as a potential conflict of interest.
