# Supplementary material for: Identifying Risk Factors for Complicated Post-operative Course in Tetralogy of Fallot Using a Machine Learning Approach
Source: Front Cardiovasc Med. 2021 Jul 22;8:685855. doi: 10.3389/fcvm.2021.685855 (PMC8339319; doi:10.3389/fcvm.2021.685855)
Supplement: Supplementary file 1 [file Data_Sheet_1.docx]

**Supplemental Material**

**Methods:**

Gradient boosted quantile regression was used to identify predictors of number of cardiac complications at the 50^th^ percentile.^19^ For each imputed dataset, we designated 80% of the data as training (for model development) and the remaining 20% as the holdout testing set (for model evaluation). On the training dataset, we fit a 10-fold cross-validated (CV) boosted regression model using 5000 gradient boosted trees and determined the optimal number of trees which minimized the cross validated root mean square error (RMSE). We used the optimal number of trees for prediction, an interaction depth of 1, and shrinkage parameter set to 0.001. Each GBM provided a relative importance for all of the variables included in the model. Relative importance scores across 10 multiply-imputed datasets were averaged and averaged relative importance scores are presented for all predictors. Model fit in the holdout data was assessed by fitting the model identified in the training dataset to predict values in the testing data.

Average CV RMSE in the training data and RMSE in the test data, averaged across the 10 imputed datasets, are presented. We compared both the average CV RMSE for the training data and average RMSE for the test data from the boosted quantile regression to corresponding average RMSE for training and test data obtained from stepwise linear regression, a commonly used approach for prediction models with a continuous outcome. Lower RMSE values indicate better model fit. Missing data were imputed using a fully conditional specification method with 10 imputations for continuous and categorical covariates using the SAS Procedure Proc MI.^18^ All analytic (regression-based or GBM) models were run individually on each imputed dataset.

**First Stage Regression models:**

To test the importance of a multi-stage modeling approach to obtain the final set of predictors, we ran each gradient boosted quantile regression with and without first-stage multivariate adaptive regression spline model (MARS) to initially select a subset of clinically meaningful predictors. Like the GBM, MARS is another powerful approach for prediction. MARS uses a series of nonlinear functions for different intervals of the predictors. We fit a negative binomial model using the MARS approach and allowed for all main effect and two-way interaction terms to be considered to find the best model, which was determined by minimizing the generalized cross validation (GCV) criterion. The relative importance of each predictor identified in the final model is available from this model, which is computed from the GCV.^21^ The boosted quantile regression models without a first-stage regression used all 56 original predictors in the model. For the two-stage approach, we first ran a MARS negative binomial regression model on each of the 10 imputed datasets and identified the top 21 predictors using the relative importance values. We decided on 50% of the predictors (N=21) since 43 of the 56 predictors were identified as important across any of the MARS models. The second stage GBMs were run using the 21 selected predictors. We obtained the relative importance for each of the variables in each GBM and then averaged the relative importance across the 10 imputed datasets to get an overall relative importance for each predictor.

**Sensitivity Analysis**

To examine if the zero-inflated distribution of the outcome could have affected our results, we also ran gradient boosted Poisson regression models on the subset of patients with at least one complication. We present the average relative importance and RMSE, across the 10 imputed datasets, for Poisson GBMs that were run using all 56 predictors and using the 21 predictors obtained from using a first stage regression from the main analysis.

**Results**

**Missing data:**

The covariates with the highest percentages of missing data are preoperative echocardiographic variables that are used to assess RV systolic and diastolic function, which were not measurable or not available in the echocardiogram: RV strain rate (50.62%), tricuspid E/e’ ratio (40.7%), tricuspid peak A velocity (40.74%), tricuspid peak e’ velocity (33.3%), and tricuspid peak S’ velocity (27.8%). Since these preoperative variables were obtained from clinically indicated echocardiograms, some patients did not have tissue Doppler or Doppler interrogation of the tricuspid inflow, or adequate RV wall images for measurement of strain rate. The remaining 59 predictors had less than 20% missing data.

| **Appendix Table 1: Descriptive statistics for type of cardiac and non-cardiac post-operative complications and timing of complications** | | | | | | | |
| --- | --- | --- | --- | --- | --- | --- | --- |
|  |  | N (%) | |  | | | |
| **Has at least one PO Cardiac Complication** | | 43 (26.54%) | | | | | |
| **Type of Cardiac Complication:** |  |  | |  | | | |
| Arrhythmia requiring Treatment |  | 22 (13.58%) | | | | | |
| Catheterization |  | 17 (10.49%) | | | | | |
| ECMO |  | 11 (6.79%) | | | | | |
| Mediastinal Exploration |  | 9 (5.56%) | |  | | | |
| Pleural Effusion requiring chest tube |  | 8 (4.94%) | |  | | | |
| Delayed sternotomy closure |  | 7 (4.32%) | |  | | | |
| Re-operation |  | 7 (4.32%) | |  | | | |
| Cardiac Arrest and CPR |  | 6 (3.70%) | |  | | | |
| Other Cardiac Procedure |  | 5 (3.09%) | |  | | | |
| Pericardial Effusion with pericardiocentesis |  | 1 (0.62%) | |  | | | |
| Pacemaker Placement |  | 1 (0.62%) | |  | | | |
|  |  |  | |  | | | |
| **First Day of Arrythmia Requiring Treatment** |  |  | |  | | | |
| 0 |  | 15 (68.18%) | | | | | |
| 1 |  | 4 (18.18%) | | | | | |
| 2 |  | 1 (4.55%) | |  | | | |
| 4 |  | 1 (4.55%) | |  | | | |
| 5 |  | 1 (4.55%) | |  | | | |
|  |  |  | |  | | | |
| **First Day of Catherization (N=17)** |  |  | |  | | | |
| 0 |  | 3 (17.65%) | | | | | |
| 3 |  | 5 (29.41%) | | | | | |
| 6 |  | 1 (5.88%) | |  | | | |
| 7 |  | 1 (5.88%) | |  | | | |
| 8 |  | 1 (5.88%) | |  | | | |
| 9 |  | 1 (5.88%) | |  | | | |
| 10 |  | 1 (5.88%) | |  | | | |
| 17 |  | 1 (5.88%) | |  | | | |
| 19 |  | 2 (11.76%) | | | | | |
| 21 |  | 1 (5.88%) | |  | | | |
|  |  |  | |  | | | |
| **First Day of Any Cardiac Complication (N=43)** |  |  | |  | | | |
| 0 |  | 28 (65.12%) | | | | | |
| 1 |  | 6 (13.95%) | | | | | |
| 2 |  | 1 (2.33%) |  | | | | |
| 3 |  | 2 (4.65%) |  | | | | |
| 4 |  | 2 (4.65%) |  | | | | |
| 7 |  | 2 (4.65%) |  | | | | |
| 8 |  | 1 (2.33%) |  | | | | |
| 19 |  | 1 (2.33%) |  | | | | |
| **At least one PO Non-Cardiac Complication, N (%)** | |  | 13 (8.0%) | | | | |
| **Type of Non-Cardiac Complication:** | |  | | |  |  | |
| G-Tube Placement | |  | | 3 (1.85%) | | |  |
| Pneumothorax Requiring Treatment | |  | | 3 (1.85%) | | |  |
| Seizure requiring Treatment | |  | | 2 (1.23%) | | |  |
| Superficial wound Infection | |  | | 2 (1.23%) | | |  |
| UTI, treated | |  | | 2 (1.23%) | | |  |
| Bronchoscopy | |  | | 1 (0.62%) | | |  |
| Laryngo/Tracheal/Bronchial Malacia | |  | | 1 (0.62%) | | |  |
| NEC, Treated | |  | | 1 (0.62%) | | |  |
| Sepsis, Treated | |  | | 1 (0.62%) | | |  |
| Vocal Chord Paralysis Confirmed | |  | | 1 (0.62%) | | |  |
| Bowel Resection | |  | | 0 (0%) | | |  |
| Dialysis | |  | | 0 (0%) | | |  |
| Diaphragm Complication | |  | | 0 (0%) | | |  |
| Diaphragm Paresis/Paralysis | |  | | 0 (0%) | | |  |
| Ladds Procedure | |  | | 0 (0%) | | |  |
| Mediastinitis | |  | | 0 (0%) | | |  |
| TE Fistula Repair | |  | | 0 (0%) | | |  |
| Tracheostomy | |  | | 0 (0%) | | |  |

PO: post-operative; ECMO: Extracorporeal Membrane Oxygenation; CPR: Cardiopulmonary Resuscitation; UTI: Urinary Tract Infection; TE: Tracheo-esophageal

| **Appendix Table 2: Boosted Poisson Models, run without first-stage regression models using sample of patients with at least one complication (N=43)** | |  |
| --- | --- | --- |
|  | |  |
| **GBM run using all variables (no first stage regression; N= 56 variables)** |  |  |
| **Predictor (ranked in descending strength):** | **Average Importance** | |
| Total CPB time | 30.85 |  |
| Age at time of surgery (months) | 20.28 |  |
| RV fractional area change (pre-operative) | 6.02 |  |
| Right pulmonary artery Z-Score | 5.67 |  |
| Admitted from Outside Hospital | 4.41 |  |
| Left pulmonary artery Z-Score | 3.74 |  |
| Total aortic cross-clamp time (min) | 3.36 |  |
| Prostaglandin use at admission | 2.80 |  |
| Partial pressure of O2 (paO2) after TOF repair | 2.78 |  |
| Oxygen Saturation (Pre-operative) | 2.76 |  |
| Global Strain- RV %(Pre-operative) | 2.25 |  |
| Aortic Arch Side= Right | 2.16 |  |
| Lowest PH on CPB | 1.78 |  |
| Outflow tract repair=RV-PA conduit | 1.76 |  |
| Feeding Status at Admission= NPO | 1.34 |  |
| Surgeon #3 (3 different surgeons) | 1.04 |  |
| Any Anomaly | 0.90 |  |
| Pulmonary valve annulus Z-Score | 0.84 |  |
| Received a Genetic Diagnosis | 0.66 |  |
| Lowest esophageal/nasal temperature in OR | 0.57 |  |
| Number of CPB runs | 0.49 |  |
| Birth weight, Kg | 0.47 |  |
| Pulmonary Valve Anatomy= Stenosis | 0.41 |  |
| Neonatal Repair (vs. repair at a later time) | 0.41 |  |
| Maternal Education= 16 or more years | 0.36 |  |
| Pulmonary arteries=Patch extended to LPA | 0.34 |  |
| Sex of Patient | 0.34 |  |
| Gestational Age (weeks) | 0.30 |  |
| Race/Ethnicity= Non-Hispanic Black | 0.23 |  |
| TOF repair access= Ventriculotomy | 0.22 |  |
| Surgeon #2 (3 different surgeons) | 0.10 |  |
| Room air use upon TOF repair admission | 0.08 |  |
| Race/Ethnicity= Hispanic | 0.07 |  |
| Endocardial Cushion Effect | 0.06 |  |
| Admitted from another unit | 0.06 |  |
| Outflow tract repair=Transannular patch | 0.03 |  |
| Lowest hematocrit on CPB | 0.01 |  |
| Pulmonary arteries=PA plasty (augmentation) | 0.01 |  |
| PFO closed at the time of TOF repair | 0.01 |  |
| No Pre-operative interventions | 0.01 |  |
| Any extracardiac malformation | 0.01 |  |
| Received a BT Shunt (pre-operative Intervention) | 0.01 |  |
| Patient was readmitted prior to this admission | 0.00 |  |
| Aorto-pulmonary collateral arteries | 0.00 |  |
| Maternal education= 13-15 years | 0.00 |  |
| Genetics consults obtained | 0.00 |  |
| Transatrial access for TOF repair | 0.00 |  |
| Coronary= Single Coronary/LAD from RCA/Dual LAD | 0.00 |  |
| Deep hypothermic circulatory arrest used at TOF repair | 0.00 |  |
| Natural conception | 0.00 |  |
| Race/Ethnicity= non-Hispanic other | 0.00 |  |
| Outflow tract repair=non-transannular patch | 0.00 |  |
| Outflow tract repair=pulmonary valvotomy | 0.00 |  |
| Continuous pulmonary arteries | 0.00 |  |
| PFO was created at the time of TOF repair | 0.00 |  |
| Pre-natal diagnosis of TOF | 0.00 |  |

| **Appendix Table 3: GBM run after first-stage regressions (N= 21 variables) in the subset with complications (N=43)** | | | |
| --- | --- | --- | --- |
|  | |  |  |
| **Predictor (ranked in descending strength)** | **Average Importance** | | |
| Total CPB Time | 35.61 | |  |
| Age at time of surgery (months) | 27.47 | |  |
| Right pulmonary artery Z-Score | 7.42 | |  |
| RV fractional area change (pre-operative) | 7.01 | |  |
| Left pulmonary artery Z-Score | 4.39 | |  |
| Total aortic cross-clamp time (minutes) | 3.93 | |  |
| Global Strain- RV % (pre-operative) | 3.80 | |  |
| Lowest PH on CPB | 3.18 | |  |
| Partial pressure of O2 (paO2) after TOF repair | 2.84 | |  |
| Lowest esophageal/nasal temperature on CPB | 1.02 | |  |
| Pulmonary valve annulus Z-Score | 0.88 | |  |
| Gestational Age (weeks) | 0.64 | |  |
| Birth weight, Kg | 0.55 | |  |
| Patch extended to LPA during TOF repair | 0.40 | |  |
| Number of CPB runs | 0.38 | |  |
| Air type= room air upon TOF repair | 0.33 | |  |
| Genetics consults obtained | 0.09 | |  |
| Lowest hematocrit on CPB | 0.03 | |  |
| Endocardial cushion defect | 0.03 | |  |
| Maternal education= 13-15 years | 0.01 | |  |
| Race/ethnicity= Non-Hispanic other | 0.00 | |  |
